# The Treatment for Tape-Worm

**Published:** 1907-09-28

**Authors:** 


### Points in Treatment.

### THE TREATMENT FOR TAPE-WORM.

There are three kinds of tape-worm common in man, each of which is derived from a different food source, and therefore prevails in one country or another according as the particular food is more or less commonly eaten there. The three worms are: (1) the *Tænia Solium*, mainly derived from the pig; (2) the *Bothriocephalus Latus*, from fish, especially the pike; (3) the *Tænia Mediocanellata*, from beef. The last-named is the commonest tape-worm in England, the Tænia Solium standing next and the Bothriocephalus Latus extremely rare.

The treatment is similar in the case of all three. It consists essentially of three stages, namely:—

1. Emptying the patient's bowel as far as possible, so as to leave little or no fæcal matter which can surround and protect the tape-worm from the drug which is to be given to kill it.
2. Administering the anthelmintic, which is essentially some preparation of male fern, and preferably the liquid extract.
3. Evacuating the dead worm.

It would sound as though the treatment is very easy; nor is it difficult if all due precautions be taken; nevertheless, it is not at all uncommon for a patient with a tape-worm to suffer from repeated recurrences. There are patients who have undergone the treatment twice or three times a year, in whom the tape-worm is still present after several years. The greater part of the worm is passed after each attempt to cure it, and for a few months no segments are passed with the stools; then they re-appear. The head of the worm esacpes death, and, remaining behind, grows in the course of three or four months into a complete tape-worm fifteen or twenty feet long again.

To kill this head it is needful to take special steps before administering the male fern. The patient may feel so well in himself that he may be loth to go to bed; but he must do so for some days. At a hospital the condition should not be dealt with at the out-patient department; the sufferer should be admitted for a few days as an in-patient. Previous to giving the male fern, the doctor orders a starvation diet combined with laxatives for two, three, or four days as the case may be; the bowel being quite empty, the male fern is given. The precise method adopted, very successfully, by Dr. J. Kingston Fowler is as follows:—

(*a*) The patient is kept in bed.

(*b*) For two, three, or in some cases four, days, according to the strength of the patient, a very low diet is given, consisting, for example, of beef-tea, 2 pints; Mason's essence, 1 tin; two rusks; and port wine, 4 oz. The only medicine given during this time is cascara sagrada, gr. ii. three times a day.

(*c*) On the third, fourth, or fifth day, as the case may be—and in most cases it will be the fourth—a purgative is given early, followed by the anthelmintic, as follows:—

| | |
|---|---|
| At 5 A.M. ... | Haustûs sennæ compositi, ℥j. |
| „ 9 A.M. ... | Extracti filicis maris, ♏xv. in capsulâ. |
| „ 9.15 A.M.... | „ „ „ ♏xv. „ |
| „ 9.30 A.M.... | „ „ „ ♏xv. „ |
| „ 9.45 A.M.... | „ „ „ ♏xv. „ |
| „ 11 A.M. ... | Haustûs sennæ compositi, ℥j. |

The worm will very likely be passed early in the afternoon. Every particle of it should be kept and examined with the greatest care, in order to detect the head. It is only when the minute head of the tapeworm has been found that a cure can be confidently asserted. If it is not found, a second course of senna and male fern should be carried out the same day; and if it should prove necessary, even a third course. Beyond this it is distinctly inadvisable to go, for the three consecutive series of doses in a state of fasting will be exhausting enough. Fortunately the whole of the worm is usually passed before the end of the day, provided the preliminary treatment of the patient by rest in bed and low diet has been rigidly adhered to; and as soon as the head has been found, full diet may be allowed at once. If the head has not been found it is best to wait three or four months before repeating the treatment. It is always possible that the head may have really been passed, though it escaped detection. If in three or four months' time the segments should unfortunately re-appear in the stools, the whole procedure should be repeated as above.

The administration of filix mass in four successive doses of 15 minims, rather than in a single dose of 1 drachm, has two advantages: first, it is conceivable that a single dose might pass by the worm without killing it, whereas this is less likely with repeated doses; secondly, the preparation has a very nauseous taste, which is not easily covered when a whole drachm is given in a draught, whereas, given in the smaller doses, it is possible to give it in capsules which are tasteless.

